# Advances in Amino Acid-Based Chemistry

**DOI:** 10.3390/ph16101490

**Published:** 2023-10-19

**Authors:** Rosanna Palumbo, Hayarpi Simonyan, Giovanni N. Roviello

**Affiliations:** 1 Institute of Biostructures and Bioimaging, Italian National Research Council (IBB-CNR), Via P. Castellino 111, 80131 Naples, Italy; rosanna.palumbo@cnr.it; 2 Institute of Pharmacy, Yerevan State University, 1 Alex Manoogian Str., Yerevan 0025, Armenia; hayarpi.simonyan@ysu.am

Numerous applications of amino acid-based compounds and peptide derivatives in different biomedicine- and nanotechnology-related fields were described in the recent scientific literature [1]. For example, glycine derivatives including glycyl-glycyl-glycine, glycyl-glycine, sarcosine, dimethylglycine, all of which were functionalized with memantine (Figure 1) moieties, were found to exert a neuroprotective effect, improving cell viability against copper- and glutamate-induced neurotoxicity [2].

A synthetic octapeptide, derived from activity-dependent neuroprotective protein (ADNP), that is able to bind to Cu^2+^ and Zn^2+^ showed peculiar crystallization properties that were influenced by the metal ions. It also exerts a neuroprotective effect due to both its metal chelating properties and its ability to interact with amyloid beta (Aβ) peptide, whose abundant deposition in the brain is famously linked to the Alzheimer’s disease [3,4].

Synthetic peptides in conjunction with growth factors can show neuroprotective properties, making them potential candidates as innovative neurodrugs. The synthetic dodecapeptide C16 administered together with the growth factor angiopoietin-1 improved functional disability and reduced neuronal cell death in animal models by protecting vascular endothelial cells, thereby inhibiting inflammatory cell infiltration and maintaining blood–brain barrier (BBB) permeability [5].

Novel cyclic peptidomimetics of the protein suppressor of cytokine signaling 3 (SOCS3) [6] were designed and synthesized for the development of novel therapeutic strategies involving the ternary protein complex formed by SOCS3 with Janus Kinase 2 and glycoprotein 130 [7].

Signs of the potential anti-metastatic activity of sugar–amino acid derivatives were discovered by the collaborative efforts of Armenian and Italian chemists who used the Amadori reaction to obtain novel synthetic conjugates and also discovered novel molecules with therapeutic potential [1].

Peptides are also useful in the field of prophylactics for the realization of vaccines. Among the others, peptide-based vaccines have recently been attracting a growing attention in the prevention and recurrence of breast cancer [8,9].

Chimeric compounds whose structures include both nucleobases and amino acid residues are known in the scientific literature as nucleoamino acids, which in turn form larger structures that are often labeled as nucleopeptides [10,11]. l-Willardiine (Figure 1) is one of several examples of a nucleoamino acid that occurs in nature, and in particular, it functions as a neurotransmitter in the human organism. Synthetic nucleoamino acids and the corresponding nucleopeptides can also be used in several biomedical and nanotechnological applications [12].

Amino acid-based materials have also been found to be capable of forming biocompatible hydrogels as new nanomaterials that can be employed in biomedical strategies. For example, synthetic derivatives of diphenylalanine were shown to form hydrogels whose structural arrangement and behavior in terms of matrix porosity, stiffness, and stability is influenced by the different formulation strategy [13].

Interestingly, peptides are also useful in cosmetics and can be used as active ingredients on sensitive skin due to their ability to interact with skin cells with high potency at low dosage and to penetrate the stratum corneum [14].

In conclusion, amino acid-derivatives and peptides are molecular tools with a vast number of applications in the field of human health, ranging from therapy to disease prophylaxis, but also find use in cosmetics and nanotechnology, as we mentioned in this work.

**List of Contributions**

1. Chayrov, R.; Volkova, T.; Perlovich, G.; Zeng, L.; Li, Z.; Štícha, M.; Liu, R.; Stankova, I. Synthesis, Neuroprotective Effect and Physicochemical Studies of Novel Peptide and Nootropic Analogues of Alzheimer Disease Drug. Pharmaceuticals 2022, 15(9), 1108; https://doi.org/10.3390/ph15091108.
2. Iavorschi, M.; Lupăescu, A.; Darie-Ion, L.; Indeykina, M.; Hitruc, G.; Petre, B. Cu and Zn Interactions with Peptides Revealed by High-Resolution Mass Spectrometry. Pharmaceuticals 2022, 15(9), 1096; https://doi.org/10.3390/ph15091096.
3. Fu, X.; Wang, J.; Cai, H.; Jiang, H.; Han, S. C16 Peptide and Ang-1 Improve Functional Disability and Pathological Changes in an Alzheimer&rsquo;s Disease Model Associated with Vascular Dysfunction. Pharmaceuticals 2022, 15(4), 471; https://doi.org/10.3390/ph15040471.
4. La Manna, S.; Leone, M.; Mercurio, F.; Florio, D.; Marasco, D. Structure-Activity Relationship Investigations of Novel Constrained Chimeric Peptidomimetics of SOCS3 Protein Targeting JAK2. Pharmaceuticals 2022, 15(4), 458; https://doi.org/10.3390/ph15040458.
5. Nordin, M.; Azemi, A.; Nordin, A.; Nabgan, W.; Ng, P.; Yusoff, K.; Abu, N.; Lim, K.; Zakaria, Z.; Ismail, N.; Azmi, F. Peptide-Based Vaccine against Breast Cancer: Recent Advances and Prospects. Pharmaceuticals 2023, 16(7), 923; https://doi.org/10.3390/ph16070923.
6. Palumbo, R.; Omodei, D.; Vicidomini, C.; Roviello, G. Willardiine and Its Synthetic Analogues: Biological Aspects and Implications in Peptide Chemistry of This Nucleobase Amino Acid. Pharmaceuticals 2022, 15(10), 1243; https://doi.org/10.3390/ph15101243.
7. Diaferia, C.; Rosa, E.; Morelli, G.; Accardo, A. Fmoc-Diphenylalanine Hydrogels: Optimization of Preparation Methods and Structural Insights. Pharmaceuticals 2022, 15(9), 1048; https://doi.org/10.3390/ph15091048
8. Resende, D.; Ferreira, M.; Sousa-Lobo, J.; Sousa, E.; Almeida, I. Usage of Synthetic Peptides in Cosmetics for Sensitive Skin. Pharmaceuticals 2021, 14(8), 702; https://doi.org/10.3390/ph14080702.

## Figures and Tables

**Figure 1:**
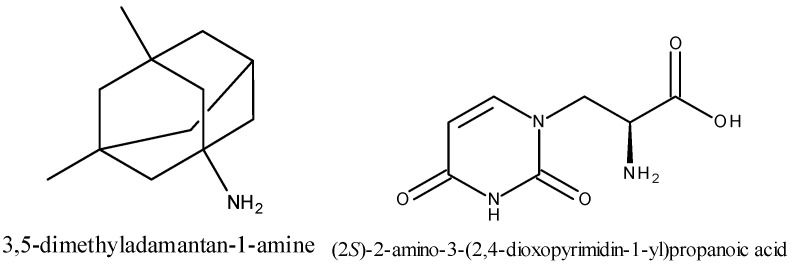
Structural representation of memantine (**left**) and l-Willardiine (**right**) with respective International Union of Pure and Applied Chemistry (IUPAC) names.
